# Author Correction: The impact of temperature on the transmissibility and virulence of COVID-19 in Tokyo, Japan

**DOI:** 10.1038/s41598-022-10651-9

**Published:** 2022-04-15

**Authors:** Lisa Yamasaki, Hiroaki Murayama, Masahiro Hashizume

**Affiliations:** 1 Department of Global Health Policy, Graduate School of Medicine, The University of Tokyo, 7‑3‑1 Hongo, Bunkyo‑ku, Tokyo, 113‑0033 Japan; 2 School of Medicine, Nagasaki University, 1‑12‑4 Sakamoto, Nagasaki, Nagasaki 852‑8523 Japan; 3 School of Medicine, International University of Health and Welfare, Kozunomori 4‑3, Narita City, Chiba 286‑8686 Japan

Correction to: *Scientific Reports* 10.1038/s41598-021-04242-3, published online 29 December 2021

The original version of this Article contained an error in the title of the paper, where the term “transmissibility” was incorrectly given as “transmissibility potential”.

The original Article and accompanying Supplementary Information file have been corrected.

